# Effectiveness and Safety of an Intravenous Immune Globulin (IVIG) Preparation in Post-exposure Prophylaxis (PEP) Against Measles in Infants

**DOI:** 10.3389/fped.2021.762793

**Published:** 2021-12-02

**Authors:** Benno Kohlmaier, Heidemarie Holzmann, Karin Stiasny, Manuel Leitner, Christoph Zurl, Volker Strenger, Michael Kundi, Werner Zenz

**Affiliations:** ^1^Department of General Pediatrics, Medical University of Graz, Graz, Austria; ^2^Center for Virology, Medical University of Vienna, Vienna, Austria; ^3^Clinical Division of Pediatric Pulmonology and Allergology, Medical University of Graz, Graz, Austria; ^4^Department of Environmental Health, Center for Public Health, Medical University of Vienna, Vienna, Austria

**Keywords:** measles post-exposure prophylaxis, intravenous immunoglobulins, measles virus specific neutralizing antibody, maternal measles-specific IgG antibodies, measles outbreak control

## Abstract

**Background:** Administration of measles virus (MV)-specific IgG as post-exposure prophylaxis (PEP) is known to effectively prevent measles. Since the introduction of active immunization against measles, the levels of MV-specific IgG antibodies in the population have dropped. Therefore, the concentration of MV-specific antibodies in immunoglobulin products derived from human plasma donors has declined as the proportion of vaccinated donors has increased. Literature on the effectiveness of PEP with current available immunoglobulins is limited. Here we examine the effectiveness of 400 mg/kg intravenous immunoglobulin (IVIG) (IgVena®, Kendrion) as PEP in infants during a measles outbreak in Austria, 2019.

**Methods:** After exposure to a highly contagious measles patient, identified infants were evaluated for eligibility for IVIG PEP. Infants were tested for measles maternal antibodies, if the result was expected to be available within 72 h after exposure. IVIG was administered to eligible infants with negative maternal IgG antibody levels (*n* = 11), infants with protective levels but result beyond 72 h (*n* = 2) and infants not tested for maternal IgG antibodies (*n* = 52). Telephone enquiries were made asking for measles infection. Effectiveness was calculated using exact logistic regression. Samples of four out of seven used IVIG batches were tested for MV-neutralizing antibody capacity.

**Results:** In 63 (96.9%) of 65 infants PEP with IVIG was administered. The parents of two infants declined IVIG PEP. None of the infants with IVIG PEP got measles or symptoms suggestive for measles, but both infants who did not receive PEP were infected. Effectiveness of IVIG PEP was calculated to be 99.3% (CI 95%: 88.7–100%). No serious adverse event of IVIG treatment was observed. The investigation on MV-neutralizing antibody capacity showed a geometric mean titer ranging from 10.0 to 12.7 IU/ml, resulting in a 1.57–2.26-fold higher concentration than postulated as minimum level for immunity.

**Conclusions:** Our findings suggest that the used IVIG preparation provided an at least non-inferior protection rate compared to IVIG preparations derived from donors before the global introduction of standard active immunization against measles.

## Introduction

Measles is a highly contagious infectious disease with high rates of complications and a high mortality (1). Especially children younger than 5 years are at high risk of developing subacute sclerosing panencephalitis (SSPE) with reported rates of 1:600–1:3,300 (2, 3). According to international guidelines, post-exposure prophylaxis with immunoglobulin (PEP) should be offered to persons without evidence of immunity against measles at high risk for complications, like infants <6 months of age, immunocompromised patients, pregnant women without evidence of immunity against measles and further persons that cannot be vaccinated for various reasons including moderate and severe acute illness. Immunoglobulins can be applied intravenously, intramuscularly and subcutaneously with contrary recommendations in Europe and the USA. Center for disease control (CDC) recommends intramuscular immunoglobulins (IMIG), when historically, IMIG has been the blood product of choice for short-term measles prophylaxis and was the product used to demonstrate efficacy for measles post-exposure prophylaxis (4). Concerns are discussed in persons who weigh >30 kg since the maximum dose by volume is 15 mL (5). Further, most IVIG preparations available in the USA are not recommended for children <2 years of age.

In contrast, the Austrian national immunization schedule and German Robert Koch institute recommend to primarily use IVIG to ensure a faster protection compared to intramuscular or subcutaneous application and is considered as less painful procedure (6, 7).

IVIG products are prepared from plasma pools from various different donated blood samples. Persons with natural immunity against measles have higher measles-specific antibody levels compared to persons with vaccination-induced immunity. Thus, the average measles-specific antibody levels in IVIG products have decreased since the introduction of the general measles vaccination programs (8).

Today, several national recommendations including Germany, Austria, USA, and Canada advise intravenous immunoglobulins (IVIG) at a dosage of 400 mg/kg (6, 9–12) but concerns regarding the decreased level of measles-specific IgG antibodies have been addressed. New Zealand raised the recommended dosage for intramuscular immunoglobulin products from 0.2 to 0.6 mL/kg, but the recommended dosage for IVIG remained unchanged (13). Recently, IVIG products available in Germany have been tested for measles-specific IgG antibodies and the authors concluded that IVIG should be further administered for post-exposure measles prophylaxis at a dosage of 400 mg/kg (9). Another report provides results from neutralization tests of 1,739 IVIG lots from a single manufacturer collected in Europe and the United States from 2013 to 2018 which showed stable measles-specific NT titers.

To evaluate effectiveness and safety of IVIG dosage recommendations detailed analysis and outbreak reports are needed, but publications on the effectiveness of measles IVIG PEP are limited. A Cochrane review showed an effectiveness of measles PEP with IVIG of 83% but 11 out of 13 included studies were published in the last century (1920–1972) whereas only two studies were published in the 21th century (2001 and 2009) (14). This might cause a not negligible uncertainty when treating clinicians have to decide, if PEP should be administered, also considering the potential harm of side effects ranging from mild symptoms to anaphylactic shock, aseptic meningitis or hemolytic anemia (15). Therefore, investigation on effectiveness of measles PEP is of high relevance especially in infants who cannot be vaccinated and who are at highest risk for measles complications including SSPE. As effectiveness data of IVIG derived from donors with active immunization against measles are limited, we describe the effectiveness of IVIG pre-parations in infants during a measles outbreak in Austria, 2019.

## Patients and Methods

In January 2019, a measles outbreak occurred with 35 laboratory-confirmed cases in Styria, a federal province of Austria. Measles virus infections were confirmed either by PCR, IgM or both. Twenty of these cases were <18 years old and eighteen of them were seen in our pediatric university hospital.

During this outbreak, multiple contacts between pediatric measles cases and other children were identified, and all persons who had either direct contact to a laboratory-confirmed measles patient or who were in the same room within 2 h after such a patient had left, were contacted by local health authorities and were asked for their measles vaccination status. Unprotected contact persons were rapidly screened for the applicability/necessity of PEP measures (vaccination or administration of IVIG).

In summary, four measles patients shared the waiting area of our Department of Pediatric and Adolescent Medicine with 30 children, eligible for IVIG PEP (29 infants and one 13-year-old girl with immunosuppression). Furthermore, five measles patients consulted five different pediatricians' practices exposing additional 40 infants eligible for IVIG PEP. One infant with measles was taken by her mother to a postnatal woman's exercise group exposing four infants eligible for IVIG PEP, and one nursery student with measles had contact to two infants eligible for IVIG PEP. In total, 76 children (75 infants aged 0–8 month and a 13-year-old girl under immunosuppressive therapy) were eligible for IVIG PEP. This included infants below 6 months of age (*n* = 63), infants > 6 months of age (i.e. eligible for active immunization) but with an exposure longer than 72 h ago (*n* = 7) and all children with a contraindication against measles vaccination (*n* = 5). All eligible children were invited for IVIG administration at our pediatrics university hospital.

**Figure 1:**
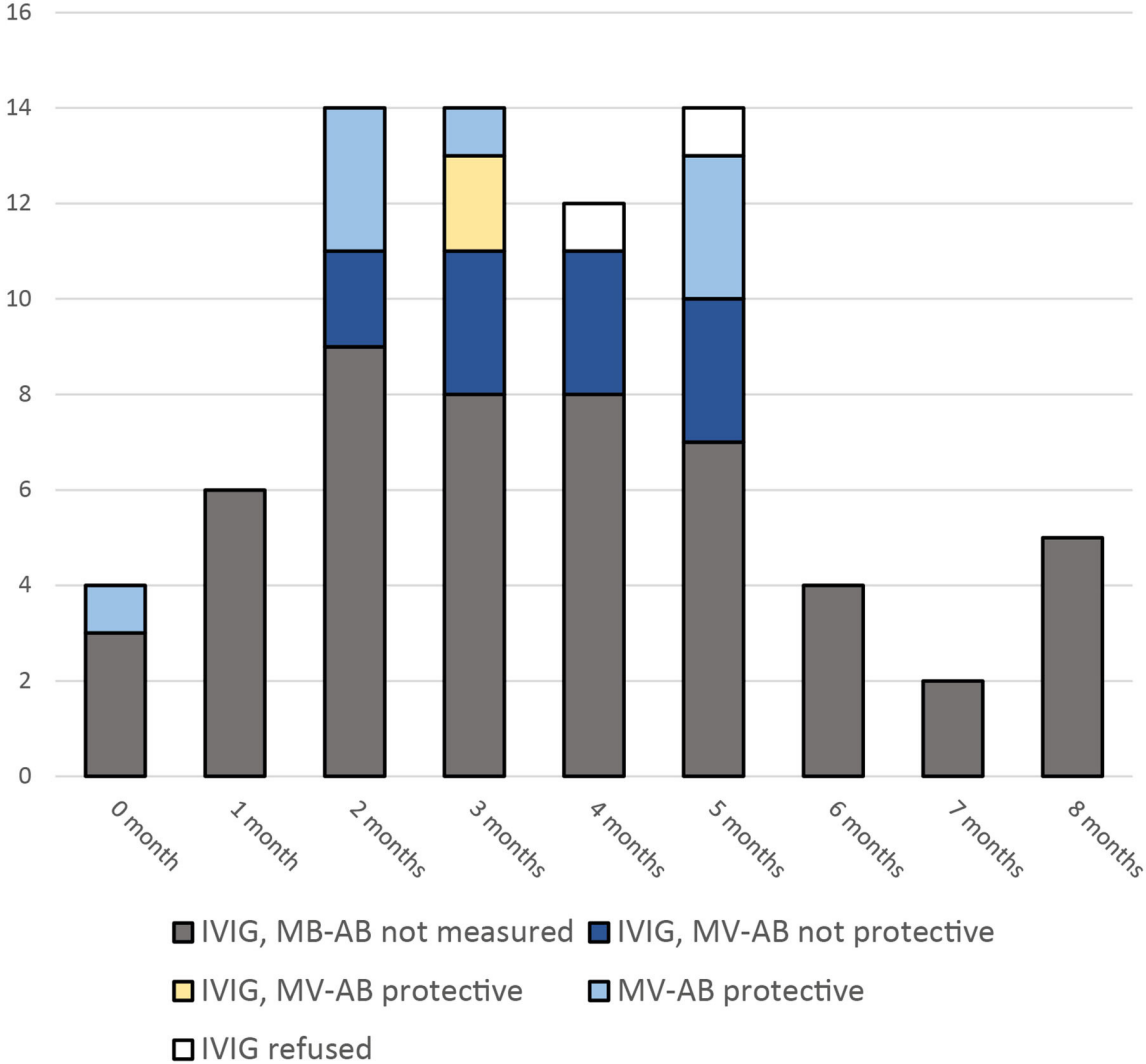
Infants eligible for PEP with IVIG after exposure to a measles patient. Measles virus specific antibodies (MV-AB) above 16.5 IU/ml were assumed to be protective. (Measured with LIAISON® Measles IgG).

Infants below 6 months of age were tested for maternal measles-specific IgG antibodies, if the result was expected to be available within 72 h after the exposure (*n* = 21). The analyses were performed by ELISA (LIAISON® Measles IgG, DiaSorin S.p.A, Italy). According to the manufacture's instruction an antibody level cutoff of >16.5 IU/ml was assumed to be protective. In untested infants, infants without timely results, and in those infants with antibody levels <16.5 IU/ml IVIG 400 mg/kg body weight (IgVena® 50g/l from Kedrion) was administered. From all 21 tested infants 10 had protective levels. Of these, two infants received IVIG, because results came too late (Figure 1).

Telephone enquiries were conducted and the parents of all children who had received IVIG treatment were called 2–3 month after the IVIG administration. We asked whether any possible side effects were observed and whether the child had developed measles or symptoms suggestive for measles (fever and maculo-papular rash).

In addition, since measles is a notifiable disease in Austria, we contacted the local health authorities to investigate whether any patient who had received PEP was recorded as measles case during this period.

Measles case definitions were used according to ECDC (16).

During the outbreak we used seven different batches of the immunoglobulins from the same manufacturer (IgVena®, Kedrion). Samples of 4 batches were sent to the Center for Virology, Medical University of Vienna for investigation on measles virus specific neutralizing antibody capacity. Each product was tested three times to derive a geometric mean titer (GMT). Calculation of IU/ml was based on the NIBSC Anti-Measles Standard (Code 97/648) for NTs (17). Calculation of achieved serum levels was performed assuming a blood volume of 85 ml/kg body weight in children <3 month and a blood volume of 75 ml/kg bodyweight in children 3 month or older.

### Effectiveness Analysis

IVIG PEP effectiveness was calculated using exact logistic regression. To balance patients who could not be contacted during telephone follow-up we hypothesized two models. The first model assumed that patients who could not be contacted but were not reported to local health authorities did not develop measles. The second model assumed that only patients who could be contacted during follow-up did not develop measles.

All infants with protective measles antibodies (*n* = 10) were excluded from the effectiveness analysis. Likewise, the only patient older than 12 months, a 13-year-old girl under immunosuppressive therapy, was excluded from calculations. She received IVIG without adverse events and didn't get measles.

### Safety Analysis

All infants were monitored for adverse events (AEs) during hospital stay. We recorded symptoms within seven days after hospital discharge according to parents' reports by the telephone follow-up. All symptoms were categorized according to Common Terminology Criteria for Adverse Events (CTCAE) Version 5.0 (18).

### Ethical Statement

The study was approved by the local Ethical Committee of the Medical University of Graz (EC-Nr. 31-364 ex 18/19). We have conducted this study consistent with the Declaration of Helsinki.

## Results

Sixty five infants were included in the effectiveness and safety analysis (Table 1). The parents of 63 infants agreed to administration of IVIG PEP, the parents of two infants refused IVIG PEP. These two patients had no direct contact to a measles case, but PEP was recommended because they had entered the waiting room of our outpatient clinic 45 min and 119 min, respectively, after a measles patient had left this area.

**Table 1:** Inclusion criteria for measles post-exposure prophylaxis (PEP) and exclusion criteria for effectiveness analysis.

Inclusion criteria for PEP
Immunosuppression (*n =* 1)
Age <6 months (*n =* 63)
Age 6–12 months and >72 h after exposure (*n =* 7)
Age 6–12 months and contraindication for vaccination (*n =* 5)
**76 children eligible for PEP**
Exclusion criteria for effectiveness analysis in infants
Age >12 months (*n =* 1)
Protective maternal antibodies (*n =* 10)
**65 infants eligible for effectiveness analysis**

Both children without PEP developed laboratory-confirmed measles (confirmed by positive measles PCR in throat swap). In the remaining 63 infants, the median delay from exposure to measles to IVIG administration was 4 days (0–6 days, IQR 2–4 day). IVIG was administered with infusion time of 6–12 h, all infants had an overnight stay.

During follow-up investigations, we were able to contact 58 (92%) parents of the 63 infants who received IVIG. None of these infants had developed measles or symptoms suggestive for measles. In addition, none of the infants was reported to local health authorities.

Assuming that all 63 patients who received IVIG did not get measles, the effectiveness of measles IVIG PEP was calculated as 99.3% (CI 95%: 88.7–100%).

Assuming that only 58 patients who received IVIG and could be reached during telephone follow-up did not get measles, the effectiveness of measles IVIG PEP was calculated as 99.2% (95% CI: 87.8–100%).

Derived from our observation that about half of infants younger than 6 months had protective maternal antibodies a further calculation was made. Even under the very conservative assumption that 50% of all infants were still protected by maternal antibodies, the effectiveness was calculated as 98.4% (95% CI: 75.3–100%).

All infants tolerated IVIG well without any adverse reaction reported during the hospital stay. One day after discharge two children developed self-limiting fever categorized as adverse event (AE) grade 1.

The investigation on MV-neutralizing antibody capacity in used IVIG products showed a GMT ranging from 10.0 to 12.7 IU/ml and 200–254 IU/g, respectively (Table 2). The batches used achieved a theoretically 941 to 1,354 mIU/ml serum level in children <3 month and 1,067–1,355 mIU/ml serum level in older children, resulting in a 1.57–2.26-fold higher concentration than postulated as minimum level for immunity lasting up to 4 weeks (9).

**Table 2:** Investigation on MV-neutralizing antibody capacity tested from samples from four different batches.

**Batches**	**Log2-NT-Titer, mean (IU/ml)**	**SD**	**GMT**	**GMT (IU/ml)**	**GMT (IU/g)**
LOT 1	9,71	0,34	839	10,0	200
LOT 2	10,05	0,24	1,057	12,7	254
LOT 3	10,05	0,24	1,057	12,7	254
LOT 4	9,71	0,34	839	10,0	200

*GMT, geometric mean titer; NT, neutralization test; SD, standard deviation*.

## Discussion

Measles post-exposure prophylaxis using IVIG is an important measure to protect infants and other vulnerable persons from measles infection and to prevent measles related complications. While there is no doubt about the usefulness of PEP, discussion about the appropriate dosage of IVIG is ongoing. We report a highly effective measles post-exposure prophylaxis using the latest recommended dosage of 400 mg/kg body weight of IVIG pre-paration derived from donors with high probability of vaccine-induced measles-specific antibodies.

In general, the effectiveness of IVIG products might be influenced by several factors. IVIG products are pooled from multiple donors to yield a wide range of antibody specificities against infectious agents (19). Individual antibody levels vary between different products and the composition of each product may vary, caused by a different natural distribution of antibody levels in the general population (9, 20). Furthermore, antibody levels of donors vary between different countries and regions caused by different endemic pathogens and vaccination schedules. The described product contains blood from multiple donors from Germany, Austria, Poland, Hungary, Czech Republic and the United States. There is no information about the proportion for each lot and about the quantity of disease-specific antibodies for each lot (15).

Besides the quality composition of IVIG products, effectiveness of PEP is influenced by delay of treatment and the probability of an infection with measles after exposure, influenced by various factors including the duration of contact, air humidity caused by seasonal variations and individual immune competence (21). Contact tracing was performed very conscientiously including an additional 2 h after a measles patient left an area to identify all children at risk but also to prevent unnecessary IVIG administration. The IgVena® product information warns about several possible side effects such as infusion reactions (e.g., flush, chills, myalgia, tachycardia, nausea and hypotension), hypersensitivity reactions (e.g., anaphylactic shock), thromboembolic events, acute kidney failure, aseptic meningitis, hemolytic anemia, neutropenia/leukopenia, acute lung injury and others (15). In our study, the administration of IVIG in infants was very safe. No infant had any event immediately after the administration and two infants had a mild self-limiting fever one day after the administration, both assessed as mild adverse events (grade 1).

The testing of the used IVIG products on MV-neutralizing antibodies showed a GMT of 839–1,057 or 200–254 IU/g, presumably resulting in a safe serum level equivalent up for 4 weeks if administered with 400 mg/kg body weight. Recent reports on MV-neutralizing antibodies in immunoglobulin products showed similar levels ranging from 148 to 436 IU/g (Germany) (9) and 240 IU/g with a standard deviation of ±134 IU/g (Australia) (13). These results confirmed an at least 1.5-fold safety margin if administered with 400 mg/kg body weight. Notable, a lower recommended dosage such as proposed in France and the UK might fall below the postulated minimum level to prevent infection (22, 23).

Incited by raised numbers of measles outbreaks in Europe and presumed lower measles-specific antibody levels in MMR-vaccinated mothers (compared to natural infection), measles-specific antibody levels in young infants were investigated with new interest. Studies concluded that most infants were susceptible to measles by 2–3 months of age (24, 25). While younger infants might benefit from a high concentration of maternal antibodies, levels halves each month, which decreases the period of protective in children from vaccinated mothers by almost 2 months (8). While the authors intention was to question the existing age recommendation for MMR vaccination, the studies also provide information about the current situation of unvaccinated infants who had contact with a measles patient. Accordingly, Robert Koch Institute suggests to test young infants (<6 month) for measles-specific antibodies after contact with a measles patient (10). In this outbreak, measuring maternal antibodies in infants eligible for PEP with IVIG was a reliable method to reduce unnecessary IVIG administration, but the feasibility was difficult, because results should be available quickly with a maximum delay of 72 h after the contact with a measles patient (10). Ten out of 21 (47.6%) tested infants had protective antibody levels and none of them developed measles although no post-exposure prophylaxis was administered in eight of them. This confirmed the suggested antibody level threshold and support the recommendation to test infants for maternal antibodies in facilities with fast laboratory results available. Further, our results emphasize that infants older than 6 months might also benefit from testing of maternal antibodies, which should be investigated in detail in future studies.

Both infants whose parents refused IVIG PEP got measles virus infection. Interestingly, one of these patients had been infected when entering the waiting room 1 h and 59 min after the respective index patient had left the room. This is in line with reports that MV has the ability to persist several hours in the air (21) and confirms current guidelines which recommend a time interval of 2 h safety interval (26, 27).

### Limitations

Due to the limited number of cases and the high protection rate among the whole cohort, it was not feasible to perform effectiveness analyses of subgroups. Several further facts might influence effectiveness such as the time interval from exposure to administration of IVIG PEP, patient's age or duration of measles exposition. The two infants who got measles after the parents refused IVIG PEP had a well-documented contact at our clinic and no further contact to other measles cases according to our contact tracing. Still, it remains a possibility that there was additional exposure to measles elsewhere in the community. Also, we cannot provide data on older infants since all eligible infants were below 9 months of age during this outbreak.

### Conclusions

Here we report the management of 75 infants eligible for PEP with IVIG after contact with a laboratory confirmed measles patient. According to our observation, the recommended dosage of IVIG with 400 mg/kg body weight was a highly effective post-exposure prophylaxis in infants.

## Data Availability Statement

The raw data supporting the conclusions of this article will be made available by the authors, without undue reservation.

## Ethics Statement

The studies involving human participants were reviewed and approved by Ethical Committee of the Medical University of Graz. Written informed consent from the participants' legal guardian/next of kin was not required to participate in this study in accordance with the national legislation and the institutional requirements.

## Author Contributions

WZ: study concept. BK: writing of manuscript draft. BK, ML, and CZ: data collection and analysis. BK, ML, and MK: statistical analysis. HH and KS: investigation on MV-neutralizing antibodies and data interpretation. All authors manuscript writing and proof reading.

## Funding

This study was financially supported by Land Steiermark (Office of the Regional Government of Styria, Department of Health Care and Science, Unit of Science and Research, Austria). The authors declare that this study received funding from Kedrion. The funder was not involved in the study design, collection, analysis, interpretation of data, the writing of this article or the decision to submit it for publication.

## Conflict of Interest

The authors declare that the research was conducted in the absence of any commercial or financial relationships that could be construed as a potential conflict of interest.

## Publisher's Note

All claims expressed in this article are solely those of the authors and do not necessarily represent those of their affiliated organizations, or those of the publisher, the editors and the reviewers. Any product that may be evaluated in this article, or claim that may be made by its manufacturer, is not guaranteed or endorsed by the publisher.
